# Expanding the ^13^C Breath Test Toolbox for Infection Detection

**DOI:** 10.1021/acscentsci.6c00515

**Published:** 2026-04-03

**Authors:** Kristin Verbeke

**Affiliations:** Translational Research in Gastrointestinal Disorders (TARGID), KU Leuven, 3000 Leuven, Belgium

## Abstract

Intravenous ^13^C-carbohydrate breath tests reliably detect bacterial infections in mice, highlighting a promising noninvasive diagnostic tool.

## Graphical abstract

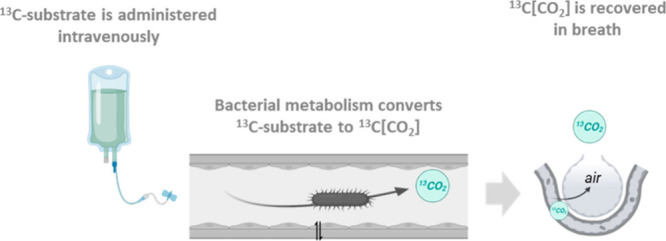

In this issue of *ACS Central Science*, Neumann, Wilson, and colleagues present a compelling proof-of-principle study in mice demonstrating the usefulness of carefully selected ^13^C-labeled carbohydrates for the *in vivo* detection of bacterial infections.^1^

The appearance of [^13^C]­CO_2_ in breath following intravenous administration of these labeled substrates reflects bacterial metabolism and therefore indicates the presence of bacterial infection.

Breath [^13^C]­CO_2_ remained undetectable in uninfected mice, but was markedly higher in mice models of myositis, bacteremia, pneumonia, and osteomyelitis. Moreover, breath [^13^C]­CO_2_ was higher in animals with more severe infection compared to those exhibiting milder disease. For statistical analysis, the authors relied on the maximal increase in delta per mil (‰) over baseline as the read-out parameter. Incorporating the entire [^13^C]­CO_2_ excretion curve and jointly analyzing all experimental conditions using linear mixed models with appropriate correction for multiple testing, rather than performing multiple *t* tests, could potentially offer a more granular view. However, such an approach would likely not alter the overall conclusions. The authors aim to further expand this promising methodological toolbox toward clinical application and suggest potential utility in other diseases including cancer or metabolic disorders.

Since the 1980s, [^13^C]­CO_2_ breath tests have been used as functional diagnostic tools in clinical gastroenterology.^2^ These tests involve the oral administration of a ^13^C-labeled substrate that undergoes the biochemical process of interest, ultimately producing [^13^C]­CO_2_ that is exhaled in breath (Figure 1). The rate at which ^13^CO_2_ appears reflects the activity or capacity of the targeted process, provided that this biochemical step is the rate-limiting step in the sequence from substrate ingestion to CO_2_ excretion. It is therefore essential to verify that none of the other steps, in particular gastric emptying or hepatic oxidation, constitutes the slowest step, especially in subgroups such as patients with delayed gastric emptying or impaired liver function.^3,4^ Breath tests are highly attractive diagnostic tools because they are noninvasive, free of radiation exposure, and safe to use in all populations, including children and pregnant women.^5^ Widely used breath tests include the ^13^C-urea test for detection of *Helicobacter pylori* in the stomach, the ^13^C-octanoic test for assessing gastric emptying, or the ^13^C-mixed triglyceride test for evaluating lipid digestion and pancreatic insufficiency.^5^ Applying ^13^C-based breath tests to diagnose bacterial infections *in vivo* expands the traditional scope significantly. The selected substrates are not metabolized by human pathways but are instead directly oxidized to [^13^C]­CO_2_ by specific bacterial species or strains. Consequently, host liver oxidation is not a potential rate-limiting step, nor is gastric emptying relevant. Indeed, to avoid confounding from commensal gut bacteria, the labeled substrates must be administered intravenously rather than orally.

**Figure 1:**
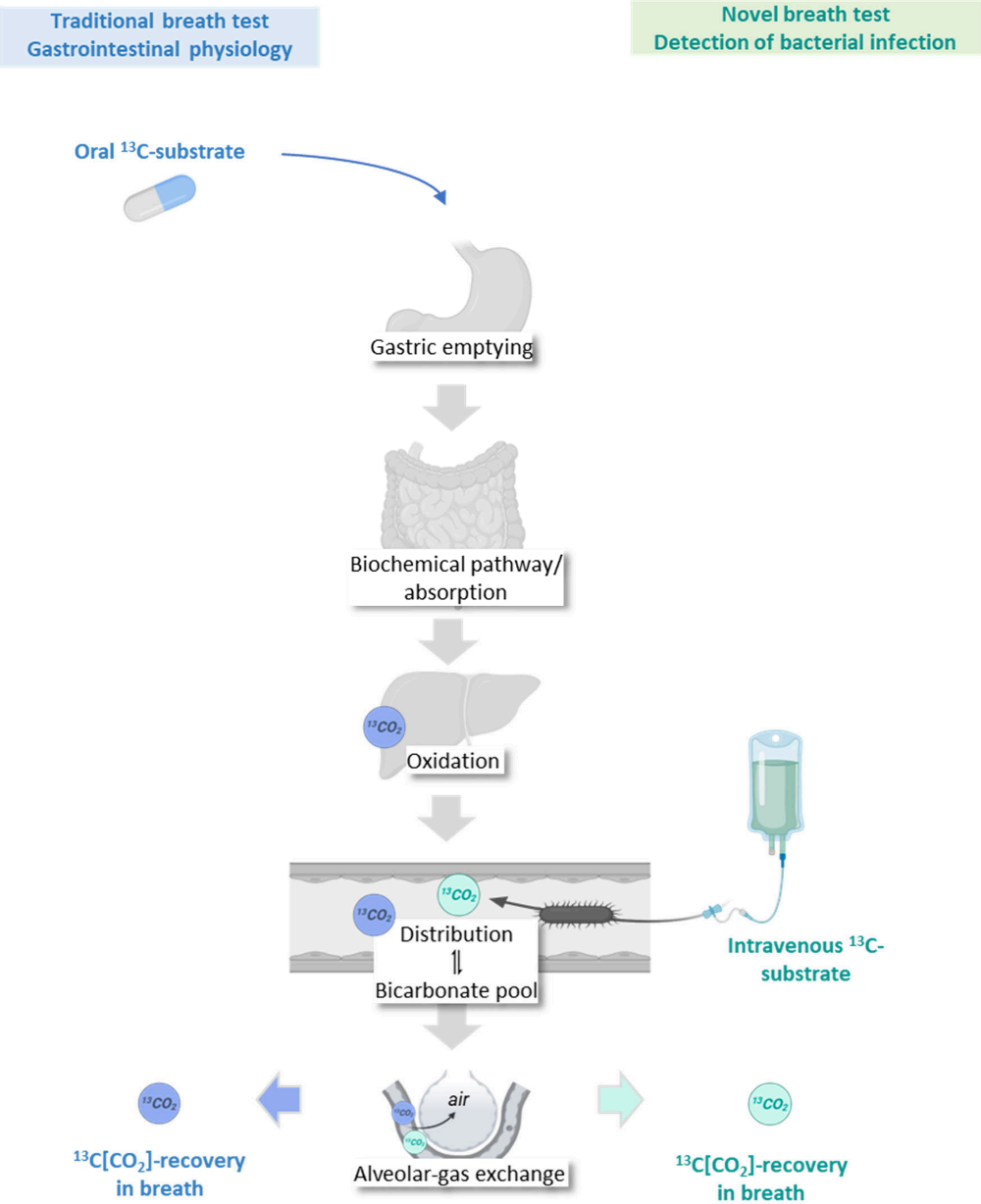
Traditional ^13^C breath tests assume that the targeted biochemical pathway is the rate-limiting step between oral administration of the labeled substrate and recovery of ^13^CO_2_ in breath. In the infection-specific approach, ^13^CO_2_ is presumed to arise exclusively from bacterial metabolism. To avoid contributions from gut microbial activity, the ^13^C-labeled substrate must therefore be administered intravenously. Figure created in BioRender.

Further development of these tests for clinical use will require methodological optimization. First, clear determination of substrate specificity and sensitivity is essential. For example, although [U–^13^C]­maltose usefully detected MRSA infection, it remains important to establish whether maltose is uniquely metabolized by *S. aureus* or whether other commensals or pathogens might similarly degrade this substrate. Establishment of pharmacokinetics, dose–effect relationships, and correlations of [^13^C]­CO_2_ with bacterial load over a broad range of bacterial concentrations may contribute to the establishment of a threshold to differentiate positive from negative tests. Second, transitioning from a qualitative test read-out (delta-overbase values) to a more quantitative assessment will enhance applicability and enable more refined analyses. Because variations in stable isotopes are typically extremely small, their abundances are conventionally expressed as δ-values (in ‰), which capture how a sample’s ^13^C/^12^C ratio differs from an international reference standard. However, these δ-values cannot be used directly for calculations. Instead, they must first be converted to absolute ^13^C-abundances, expressed as atom percent.^2^ Traditional ^13^C breath tests typically convert measured δ-values into percent of administered dose recovered, thereby accounting for the host CO_2_-production which dilutes the [^13^C]­CO_2_ signal. Since postprandial CO_2_ production, and thus the degree of dilution, is higher, more variable, and dependent on meal composition, quantitative breath tests require standardized fasting conditions. Complete tracer recovery cannot be achieved, since a considerable proportion of the generated [^13^C]­CO_2_ enters the body’s large bicarbonate pool, delaying or preventing its immediate appearance in breath.^6^

Among the challenges highlighted by the authors in developing a human-applicable test, a major regulatory obstacle is the requirement that all substrates used in human studies and eventual clinical applications be manufactured under good manufacturing practice (GMP) conditions.

At present, the number of ^13^C-labeled substrates produced according to GMP standards is extremely limited, and the associated costs are correspondingly high. Progress in this field therefore depends on sufficient growth in demand from the clinical and translational research community to incentivize suppliers to produce these substrates at GMP-quality and at more accessible prices.

In conclusion, the use of intravenously administered ^13^C-labeled substrates for detecting bacterial infections *in vivo* represents an exciting expansion of the traditional ^13^C breath test portfolio. The compelling preclinical data suggest that this approach could evolve into a powerful, noninvasive diagnostic tool capable of identifying a broad spectrum of infections with high specificity. As methodological refinements continue and interest in clinical translation grows, this promising technology has the potential to open new avenues not only in infectious disease diagnostics but perhaps also in the detection of other pathological processes such as cancer or metabolic dysfunction.
